# Glucomannan- and glucomannan plus spirulina-enriched pork affect liver fatty acid profile, LDL receptor expression and antioxidant status in Zucker fa/fa rats fed atherogenic diets

**DOI:** 10.1080/16546628.2017.1264710

**Published:** 2016-12-20

**Authors:** Laura González-Torres, Cátia Matos, Miguel Vázquez-Velasco, Jorge A. Santos-López, Iria Sánchez-Martínez, Camino García–Fernández, Sara Bastida, Juana Benedí, Francisco J. Sánchez-Muniz

**Affiliations:** ^a^ Departamento de Nutrición y Bromatología I (Nutrición), Facultad de Farmacia, Universidad Complutense de Madrid, Madrid, Spain; ^b^ Departmento de Farmacología, Facultad de Farmacia, Universidad Complutense de Madrid, Madrid, Spain; ^c^ Departmento de Nutrición, Facultad de Veterinaria, Universidad de León, León, Spain

**Keywords:** Glucomannan, spirulina, functional meats, oxidative stress, fatty acid profile, hypercholesterolemia

## Abstract

We evaluated the effects of glucomannan or glucomannan plus spirulina-restructured pork (RP) on liver fatty acid profile, desaturase/elongase enzyme activities and oxidative status of Zucker fa/fa rats for seven weeks. Control (C), glucomannan (G) and glucomannan/spirulina (GS)-RP; HC (cholesterol-enriched control), HG and HGS (cholesterol-enriched glucomannan and glucomannan/spirulina-RP) experimental diets were tested. Increased metabolic syndrome markers were found in C, G and GS rats. Cholesterol feeding increased liver size, fat, and cholesterol and reduced antioxidant enzyme levels and expressions. Cholesterolemia was lower in HG and HGS than in HC. GS vs. G showed higher stearic but lower oleic levels. SFA and PUFA decreased while MUFA increased by cholesterol feeding. The arachidonic/linoleic and docosahexaenoic/alpha-linolenic ratios were lower in HC, HG, and HGS vs. C, G, and GS, respectively, suggesting a delta-6-elongase-desaturase system inhibition. Moreover, cholesterol feeding, mainly in HGS, decreased low-density-lipoprotein receptor expression and the delta-5-desaturase activity and increased the delta-9-desaturase activity. In conclusion, the liver production of highly unsaturated fatty acids was limited to decrease their oxidation in presence of hypercholesterolaemia. Glucomannan or glucomannan/spirulina-RP has added new attributes to their functional properties in meat, partially arresting the negative effects induced by high-fat-high-cholesterol feeding on the liver fatty acid and antioxidant statuses.

## Introduction

The Zucker fa/fa rat is an animal model predisposed to obesity and metabolic syndrome. This strain is very sensitive to hypercaloric and hyperlipidic diets [1] and therefore is prone to hypercholesterolemia. Hypercholesterolemic diets can affect the expression levels of antioxidant enzymes [2,3] while free radicals caused by oxidative stress can lead to cell and tissue damage possibly resulting in cancer or cardiovascular disease [4,5].

Meat and meat products are essential in a balanced and optimal diet as they contain a large number and amount of nutrients such as proteins, minerals, vitamins and fats [6]. Although meat in the diet has been indicative of health and prosperity for centuries, in recent decades its content in saturated fatty acids has been associated with cardiovascular disease (CVD), mainly with an increase in low density lipoproteins (LDL) [7].

Food technology is responsible for quantitative and qualitative modifications in meat and meat matrices creating functional meat products. Our group has done extensive work on restructured pork (RP) enriched with different ingredients such as algae, omega-3 fatty acids and minerals [8,9]. Those RP must be able to provide a beneficial effect on one or more selective body functions, improving health and wellness while reducing the risk of disease [6]. Functional ingredients such as *Spirulina platensis* or *Konjac glucomannan* are used in these modifications. *Spirulina* is a microalga rich in minerals, antioxidant compounds (mainly carotenoids and phycocyanin) and a biliprotein pigment with hypocholesterolemic activity [10]. *Konjac glucomannan* is an indigestible polysaccharide extracted from the tubercle of *Amorphophallus konjac* [11]. Some studies suggest the usefulness of *Konjac glucomannan* in the treatment of obesity, dyslipidemia and diabetes [12]. Some studies also suggest that glucomannan promotes satiety [13], has a prebiotic effect [14] and acts as an immunomodulator [15] or hypocholesterolemic [16]. Glucomannan has been added to squid surimi and promising results on cholesterolemia and antioxidant status have been found in cholesterol-fed rats, mostly when associated with spirulina [17]. However, controversial results were observed in some inflammatory biomarkers [17,18]. We very recently published results on the effects of glucomannan and glucomannan/spirulina-enriched RP in atherogenic diets on lipids and lipoproteins [19]. Both RP were able to mitigate the cholesterolemic effects of dietary cholesterol by decreasing the level of atherogenic lipoproteins, especially in cholesterol-fed animals.

Based on previous research done by our group, we hypothesized that at hypercholesterolemic status the liver increases the esterification of free-cholesterol with oleic acid [20]. Body weight gain in cholesterol-fed animals was also reduced probably due to a hormone-sensitive lipase (HSL) increase accelerating the release of free fatty acids from adipose tissue [19]. Nevertheless, either liver fatty acid profile or LDL receptor (*Ldlr*) expression has been tested in fa/fa rats in an attempt to discover a potential mechanism linking all these results.

The biological mechanisms linking estimated desaturase activity with obesity and diabetes mellitus type 2 (T2DM) are still unclear. Delta-6 desaturase is the rate-limiting step along the PUFA pathway which includes arachidonic acid, a precursor from eicosanoids that may act as an inflammation mediator [21]. Increased delta-5-desaturase (D5D) activity has been associated with high plasma concentrations of eicosapentaenoic (EPA) and docosahexaenoic (DHA) acids, which have anti-inflammatory properties and triglyceride-lowering effects [22]. Stearoyl-CoA desaturase-1 (SCD1) is the rate-limiting enzyme in the synthesis of MUFAs [23]. Genetic variants of SCD1 have been associated with dyslipidemia, inflammation and increased liver fat in T2DM [24].

The relevance of the present work lies in the possible application of Glucomannan- and spirulina-RP to improve the liver fatty acid and antioxidant status profiles of hypercholesterolemic rats. Thus, we hypothesized that adding glucomannan or glucomannan *plus* spirulina to RP would ameliorate the negative effect of cholesterol feeding on both profiles. As no studies have been performed to evaluate these changes, the aims of this paper were to evaluate the effect that glucomannan/glucomannan *plus* spirulina-RP consumption in fa/fa rats have on main liver characteristics: (a) size, fat and cholesterol content; (b) fatty acid composition and *Ldlr* expression; (c) desaturases and desaturase-elongase activities; (d) antioxidant status based on the quantification of activities and expressions of several antioxidant enzymes.

## Material and methods

### Diet preparation and experimental design

This study was approved by the Spanish Science and Technology Advisory Committee (project AGL 2008–04892-C03-02 and Consolider Ingenio 2010, CSD 2007–00016) and by the ethics committee of the Complutense University of Madrid (Spain). All experiments were performed in compliance with Directive 2003/65/EEC of the European Parliament and of the Council of 22 July 2003 on the protection of animals used for scientific purposes. A total of thirty-six growing male Zucker fa/fa rats with an initial body weight of approximately 120 g were obtained from Harlan Laboratories Models (Harlan, SL, Barcelona, Spain). The animals were housed individually in metabolic cells in a temperature-controlled room (22.3 ± 1.9 °C) with a 12 h light-dark cycle. The rats were fed commercial pellets (Panlab, Barcelona, Spain) for one week to adapt to environmental conditions and then distributed into six groups of six animals each according to their average body weight. Rats were fed the experimental diets for 7 weeks. Food consumption was measured daily and body weight once per week. Tap water was provided *ad libitum* over the experimental period with an average consumption of 10 mL/100 g body weight/day. In order to avoid inter-assay variations that could affect the comparison of data from the different groups, at the end of the experiment fasting rats were taken one at a time from each of the six groups, anesthetized with pentobarbital (45 mg/kg) and euthanized by extracting blood from the descending aorta with a syringe. Then, plasma was separated by centrifugation at 2000 *g* for 10 min. Livers were collected and weighted immediately after blood extraction.

Glucomannan (*Amorphophallus konjac*; powder from Guinama, S.L.U., Valencia, Spain; 22.5 g/kg diet) and Spirulina (*Arthrospira maxima*; micronized powder from Arkopharma, Madrid, Spain; 3.0 g/kg diet) were used in the preparation of diets and calculated according to previous studies from Hozumi et al. (1995), Bermejo-Bescós et al. (2008), and Ou et al. (2013). Pork was purchased at a local market, then freeze-dried and mixed with the rest of the ingredients until fully homogenous to form the restructured pork (RP). The following six semi-synthetic experimental diets were prepared: control diet with no added cholesterol (C) was composed of a homogeneous mixture of 85% rodent diet (AIN-93M purified rodent diet; Dyets, Inc., Bethlehem, PA, USA) [25] and 15% freeze-dried RP (with 15% microcrystalline cellulose); glucomannan normocholesterolaemic diet (G) consisted of a mixture of AIN-93M no.102635 feed (85%) and freeze-dried glucomannan-enriched RP (15%); glucomannan *plus* spirulina normocholesterolaemic diet (GS) consisted of a mixture of AIN-93M no.102635 feed (85%) and freeze-dried glucomannan *plus* spirulina-enriched RP (15%); cholesterol-enriched control diet (HC) was identical to diet C but with 2.43% cholesterol (95–98% purity) and 0.49% cholic acid (98% purity) replacing an equal amount of corn starch (AIN-93M no. 102,636 diet); cholesterol-enriched glucomannan diet (HG) was the G diet enriched with cholesterol (2.43%) and cholic acid (0.49%); the cholesterol-enriched glucomannan *plus* spirulina diet (HGS) consisted of the GS diet enriched with cholesterol (2.43%) and cholic acid (0.49%). The amount of cholesterol and cholic acid added to diet was based on previous studies [26]. Diets were designed to cover rat nutritional requirements. Details of diet composition can be found in the **Supplementary Table**.

### Determination of fatty acid profile in liver

Liver fat was extracted following the Folch method [27]. 50 mg of fat were characterized in the form of methyl esters in capillary gas chromatography in accordance with the procedure followed by Carrapiso et al. [28]. Gas chromatography (GC) was performed using an HP 5890II (Hewlett Packard) equipped with a cold on-column injector and a flame ionization detector (FID). Individual fatty acid methyl esters (FAMEs) were separated with a 30 m × 0.53 mm capillary column coated with FFAP-TPA stationary phase (1 mm thickness). The GC conditions were as follows: oven temperature 220 °C isothermal for 30 min, injector and detector temperature 230 °C. The flow rate of the carrier gas (nitrogen) was 2.6 ml/min and 0.1 ml of solution was injected. Chromatographic data were recorded and integrated in an HP computer (HP Vectra QS/20) using a Hewlett Packard ChemStation A.01.14 software. Fatty acid methyl esters were identified by comparison of retention times with standards. Peak areas were corrected using FID response factors estimated from standards. The concentrations of FA (g/100 g methyl ester) were calculated by means of the internal standard (tridecanoic acid methyl ester) and sample weight. Some products-to-precursor ratios were used as surrogated indices of enzymes desaturase [24] or desaturase-elongase activities [29], using the following formulas:

Delta-6-elongase-desaturase:

(a) Docosahexahenoic acid/linolenic acid (C22:6 n-3/C18:3 n-3)

(b) Arachidonic acid/linoleic acid (C20:4n-6/C18:2 n-6)

Stearoyl-CoA activity:

Palmitoleic acid/palmitic acid (C16:1 n-7/C16:0)

Oleoyl-CoA activity:

Oleic acid/stearic acid (C18:1 n-9/C18:0)

Delta 5 desaturase activity:

Arachidonic acid/eicosatrienoic acid (C20:4 n-6/C20:3 n-6)

Delta-6 desaturase activity:

Gamma-linolenic acid/linoleic acid (C18:3 n-6/C18:2 n-6)

### Western blotting and protein levels

Total liver protein lysates were obtained and separated in 10% sodium dodecyl sulfate-poly-acrylamide gel electrophoresis (SDS-PAGE). Gels were then blotted onto PVDF Amersham Hybond-P membranes (GE Health-care, Buckinghamshire, UK) and incubated with their corresponding antibodies (anti-β-actin (A2228), anti-catalase (C0979), anti-superoxide dismutase (S2147) from Sigma–Aldrich, St. Louis, Missouri, USA; anti-glutathione peroxidase (Ab59546), from Abcam, Cambridge, UK; anti-glutathione reductase (sc-32886) from Santa Cruz Biotechnology, Dallas, Texas, USA). β-actin was used as loading control. Blots were developed by enhanced chemiluminescence using an Amersham ECL Plus Western Blotting Detection Reagent (GE Health-care, Buckinghamshire, UK) according to manufacturer’s instructions.

### RNA extraction

Total RNA was isolated from 100 mg of liver using Trizol (Invitrogen, Carlsbad, CA, USA) according to the manufacturer’s instructions.

### RNA degradation

Isolated RNA samples were treated with DNase I RNase-free, DNase treatment and removal reagents (Thermo Fisher Scientific, Waltham, Massachusetts, USA) to remove any contamination with genomic DNA. To check the potential RNA degradation the methodology published in *Sample preparation techniques in analytical chemistry* [30] was followed, we performed an agarose gel (1.3%) to visualize the 18S and 28S bands and determine whether degradation have occurred during the isolation procedure, or if the RNA was degraded in the tissue prior to RNA isolation.

### RNA integrity and quantification

To verify the integrity and to quantify RNA, the Fleige and Pfuffl protocol [31] with slight modifications was followed. This protocol permitted us to confirm the contaminants absence of RNA with proteins, salts, or organic reagents such as phenol or chloroform. Thus, we performed a quantity and integrity assessment using a UV/VIS spectrophotometer at multiple wave lengths: at 260 nm for nucleic acids; 270 nm for possible contaminants, mainly phenol derivates; 280 nm for proteins; and 310 nm for background and presence of salts or organic solvents. The 260 nm/280 nm ratio was used to verify the RNA integrity, and the 260 nm/310 nm ratio to verify the purity and the extraction performance. A 260 nm/280 nm ratio greater than 1.9 was considered an acceptable indicator of good RNA quality.

### Gene expression and quantification by RT-PCR

Total RNA of each sample (1 µg) was reverse-transcribed to first-strand complementary DNA (cDNA) using a Revert Aid H minus first strand cDNA synthesis kit (Thermo Fisher Scientific, Waltham, Massachusetts, USA).

Relative superoxide dismutase (Cu/Zn-SOD, Mn-SOD), catalase (CAT), glutathione peroxidise (GPx), glutathione reductase (GR) and *Ldlr* mRNA levels were quantified using RT-PCR with a LightCycler Real Time PCR Detection System (Roche Diagnostics, Indianapolis, Indiana, USA), using a SYBR® Green (Biotools, Madrid, Spain) as binding dye.

The PCR parameters were as follows: preincubation at 95 °C for 10 minutes followed by 40 cycles of denaturation at 95 °C for 5 s, with an annealing temperature dependent of each couple primer (55 °C for Mn-SOD; 56 °C for Cu/Zn-SOD, 67 ºC for *Ldlr* and 60 ºC for CAT, GPx and GR), extension 72 °C for 30 s. Melting curve 95 °C–65 °C–95 °C and cooling 40 °C.

For each gene, primers were designed using average melting temperature settings near to 60 °C and Amplicon product sizes ranging from 70 to 180 bp. Primers sequences were obtained from GenBank of NCBI as follows:

Cu/Zn-SOD sense: 5ʹ GCCGTGTGCGTGCTGAA 3ʹ

Cu/Zn-SOD antisense: 5ʹ TGACGATGCCGTGCTGCATG 3ʹ

Mn-SOD sense: 5ʹ GACAAACCTGAGCCCTAAGGG 3ʹ

Mn-SOD antisense: 5ʹ CTTCTTGCAAACTATG 3ʹ

CAT sense: 5ʹ ATCAGGGATGCCATGTTGTT 3ʹ

CAT antisense: 5ʹ GGGTCCTTCAGGTGAGTTTG 3ʹ

GR sense: 5ʹ TCA CTG CTC CGC ACA TCC 3ʹ

GR antisense: 5ʹCTC AAC ACC GCC AGC GTT CTCC 3ʹ

GPx sense: 5ʹCCAATCAGTTCGGACACCAG 3ʹ

GPx antisense: 5ʹAAAGTTCCAGGCAATGTCGT 3ʹ

*Ldlr* sense: 5ʹ CTGTATTCACGGTAGCCGCC 3ʹ

*Ldlr* antisense: 5ʹ TGGGTCACATTGATGCAGCC 3ʹ

β-Actin sense: 5ʹ TACAACCTCCTTGCAGCTCC 3ʹ

β-Actin antisense: 5ʹGGATCTTCATGAGGTAGTCAGTC 3ʹ

All sample mRNA levels were normalized to their values of β-actin and the results expressed as fold changes of threshold cycle (Ct) value relative to controls using the 2^−ΔΔCt^ method [32].

### Determination of glutathione and redox index

The determination of oxidized (GSSG) and reduced (GSH) glutathione was performed according to the Hissin and Hilf method [33]. This method is based on the ability of GSH to react with a fluorescent dye (o-phthaldialdehyde, OPT). Liver tissue was homogenized in phosphate-EDTA buffer (0.1M sodium phosphate and 0.005M EDTA, pH 8) at 100 mg/mL, with the addition of 10 µL/mL of perchloric acid. It was then centrifuged at 10,000 rpm for 10 min at 4 °C. Fluorescence was measured at 350 and 420 nm (excitation and emission wavelengths, respectively). Concentrations were calculated using a standard GSH and GSSG curve. Results were expressed as μg glutathione/mg tissue. The calculated redox index (RI) indicates the antioxidant status of tissue obtained as follows: RI = GSSG/(GSH + GSSG).

### Plasma cholesterol, triglyceride, glucose levels and TyG index

Total cholesterol was measured by following spectrophotometrically the formation of quinonimine at 505 nm. Triglyceride and glucose levels were measured by following spectrophotometrically the formation of quinone with their corresponding lipases (lipoprotein lipase and phospholipase respectively) at 505 nm. All reactions were determined from 10 µL of plasma using standard enzymatic colorimetric kits from SPINREACT (San Esteve de Bas, Girona, Spain) according to manufacturer´s instructions. Intra- and inter-assay variation coefficients for glucose, triglycerides and cholesterol were < 5%.

The triglyceride-glucose index (TyG) was used to establish the existence of insulin resistance. This index has been considered a surrogate index of the HOMA index [34] and was calculated according to the following formula:

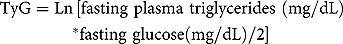

### Statistical analyses

All experiments were performed in triplicate. Statistical analyses were performed using the SPSS version 19.0 statistical analysis package (SPSS, Inc., Chicago, IL, USA). Results were expressed as means and standard deviations. A two-way ANOVA (cholesterol and diet) was used. Pairwise comparisons of diet responses between groups were made employing the Bonferroni test. Where variances were assumed to be unequal, the T2 of the Tamhane *post hoc* test was applied. The effect of cholesterol consumption was evaluated using an unpaired Student’s *t* test. Differences in growth rate induced by diets were assessed by the ANOVA test (SAS statistical packet 9.2).

## Results

**Figure 1.**
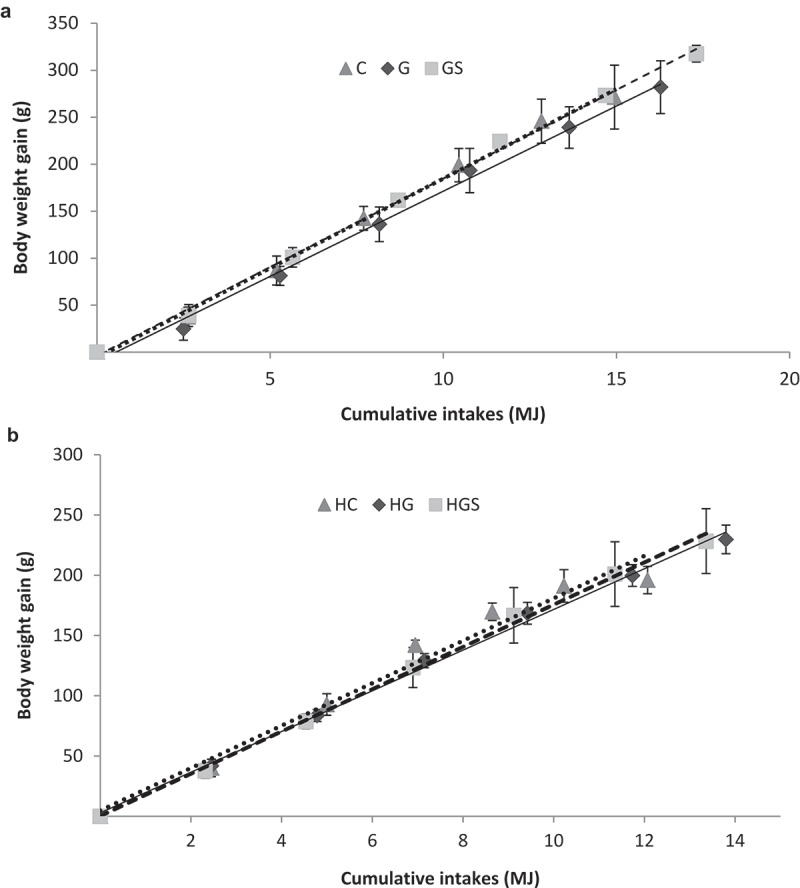
(a) Growth rates (body weight gain (g)/food consumption (MJ)) in rats fed the control, glucomannan- and glucomannan *plus* spirulina-enriched pork experimental diets. Y = (slope with their standard error) X + (intercept with their standard error). Where Y is the body weight gain and X is the food consumption. Control-RP (C): Y = (19.07 ± 3.38) X + (−5.94 ± 0.001); *r* ^2^ = 0.996 (*p *< 0.001); β = 0.998. Glucomannan-RP (G): Y = (18.13 ± 3.22)X + (−9.94 ± 0.001); *r* ^2^ = 0.995 (*p *< 0.001); β = 0.990. Glucomannan *plus* spirulina-RP: Y = (18.86 ± 1.76) X + (−3.73 ± 0.001); *r* ^2^ = 0.998 (*p *< 0.001); β = 0.998. Mean values were significantly different (ANCOVA test) for C *vs*. G. C *vs*. GS. and G *vs*. GS (all *p *< 0.05). (b) Growth rates in rats fed the control. Glucomannan- and glucomannan *plus* spirulina-RP experimental diets with supplementary cholesterol. Hypercholesterolemic control-RP (HC): Y = (17.62 ± 3.28) X + (4.69 ± 0.001); *r* ^2^ = 0.973 (*p *< 0.001); β = 0.987. Hypercholesterolemic glucomannan-RP (HG): Y = (16.88 ± 2.01) X + (2.77 ± 0.001); *r* ^2^ = 0.997 (*p *< 0.001); β = 0.995. Hypercholesterolemic glucomannan *plus* spirulina-RP (HGS): Y = (17.54 ± 2.94) X + (0.15 ± 0.001); *r* ^2^ = 0.998 (*p *< 0.001); β = 0.989. Mean values were significantly different (ANCOVA test) for HC *vs*. HG and HC *vs*. HGS (both *p *< 0.05). RP: restructured pork.

Figures 1(a,b) show the relationship between cumulative feed intake and body weight gain, together with linear adjustment parameters (*r* ^2^, slope and intercept, and their respective mean errors). All growth rate curves showed linear adjustments. Growth rate of C and HC rats significantly differ from those of their G and GS and HG and HGS counterparts, respectively. Supplementary dietary cholesterol significantly affected growth curves (HC *vs*. C, HG *vs*. G, and HGS *vs*. GS).

Table 1 shows that feed intake was significantly affected by the interaction of RP and cholesterol. Supplementary cholesterol and the type of RP influenced significantly weight gain, feed intake and feed conversion rate. The *post hoc* study showed that HC, HG, HGS *vs*. C, G, GS groups showed lower growth and lower feed intake while HC and HG presented lower feed conversion ratio than C and G. The addition of spirulina to glucomannan increased weight gain, final body weight and feed intake when compared to C animals. HG rats displayed higher feed intake than HC and HGS.

**Table 1.** Effects of glucomannan or glucomannan *plus* spirulina-RP on final weight, liver fat, total cholesterol and glucose of cholesterol-fed fa/fa rats.

	Cholesterol addition	Control RP (C/HC)	Glucomanan-RP (G/HG)	Glucomanan *plus* Spirulina-RP (GS/HGS)	ANOVA		
					RP effect	Cholesterol effect	RP*Cholesterol interaction
Weight gain (g)	No	285.6 ± 39.1 ^a^	314.7 ± 28.5 ^ab^	349.4 ± 11.7 ^b^	0.002	<0.001	0.60
	Yes	210 ± 23.3**	240.3 ± 17.9***	250.7 ± 43.0**			
Feed conversion ratio^1^	No	0.23 ± 0.02	0.25 ± 0.01	0.26 ± 0.01	0.005	<0.001	0.47
	Yes	0.20 ± 0.02*	0.21 ± 0.02***	0.23 ± 0.04			
Feed intake (g)	No	1239 ± 69.9 ^a^	1279 ± 91.0 ^ab^	1344 ± 39.7 ^b^	<0.001	<0.001	0.005
	Yes	1058 ± 59.7 ^a***^	1129 ± 80.4 ^b*^	1089 ± 32.1 ^a***^			
Liver weight (g)	No	11.8 ± 1.2	13.4 ± 2.8	15.0 ± 0.7	0.55	<0.001	0.11
	Yes	28.2 ± 3.2***	29.7 ± 2.4***	25.5 ± 6.7**			
Liver fat (%)	No	13.6 ± 7.6	10.2 ± 3.1	11.17 ± 2.9	0.52	˂0.001	0.14
	Yes	19.4 ± 5.2	24.4 ± 5.8*	27.9 ± 6.4*			
Liver cholesterol (mmol/g)	No	7.9 ± 3.6	12.5 ± 4.2	12.6 ± 3.7	0.073	0.015	0.71
	Yes	12.9 ± 4.3	15.5 ± 4.3	19.3 ± 6.3			
Plasma cholesterol (mmol/L)	No	4.4 ± 0.9	4.3 ± 0.4	4.7 ± 0.9	˂0.001	˂0.001	˂0.001
	Yes	25.6 ± 8.4^a***^	10.8 ± 2.1^b***^	6.1 ± 3.6^b^			
Plasma glucose (mmol/L)	No	19.3 ± 1.9	19.7 ± 4.1	18.9 ± 3.4	0.99	˂0.001	0.83
	Yes	12.8 ± 3.5**	12.4 ± 7.9	13.8 ± 4.8			
Plasma triglycerides (mmol/L)	No	1.69 ± 1.08	1.75 ± 0.77	1.74 ± 0.47	0.055	0.12	0.022
	Yes	2.95 ± 0.79 ^a*^	1.84 ± 0.27 ^b^	0.96 ± 0.41 ^c*^			
TyG index^2^	No	10.0 ± 0.6	10.1 ± 0.4	10.1 ± 0.3	0.085	0.025	0.026
	Yes	10.3 ± 0.3^a^	9.7 ± 0.5^ab^	9.1 ± 0.7^b*^			
*Ldlr* expression	No	100.0 ± 44.8	110.0 ± 78.5	92.0 ± 28.8	0.56	<0.001	0.52
	Yes	28.7 ± 23.0^a**^	0.9 ± 0.7^b**^	0.2 ± 0.2^b***^			

Values (mean ± SD, *n* = 6) in the same row bearing different letters were significantly different (*p *< 0.05; Bonferroni or T2-Tamhane tests). Values for the same diet but with different cholesterol content bearing asterisks were significantly different (*p < 0.05, **p < 0.01, ***p < 0.001). ^1^Feed conversion ratio = weight gain (g/day)/food intake (g/day); ^2^TyG index: Ln (fasting triglycerides (mg/dL) x fasting glucose (mg/dL) /2). C/CH, control-RP with or without added cholesterol; G/HG, glucomannan-RP with or without added cholesterol; GS/HGS, glucomannan plus spirulina-RP with or without added cholesterol.

Liver weight, fat and cholesterol content together with the hepatosomatic index were significantly affected by cholesterol feeding but not by the RP-type or the cholesterol-RP type interaction. Liver fat was significantly higher in groups HG, HGS vs. G, GS. Plasma cholesterol increased in HC and HG *vs*. C and G groups. HG and HGS rats had lower plasma cholesterol levels than those of the HC group. Plasma glucose was lower in HC *vs*. C group. Plasma glucose was lowered by cholesterol feeding and HC presented significantly lower glycemia than C rats. The TyG index was modified by the RP-cholesterol interaction and cholesterol effect. TyG was lower in HGS compared to HC rats.

*Ldlr* expression was significantly reduced by cholesterol feeding. Significant differences were observed in HC, HG and HGS rats *vs*. C, G and GS rats, respectively. Although no significant differences were observed between the non-cholesterol-fed groups, *Ldlr* expression was significantly lower in HG and HGS than HC rats. Table 2 shows that the percentage of arachidonic acid in liver was significantly affected by the type of RP* cholesterol interaction. The ‘cholesterol factor’ significantly affected the percentages of palmitic, stearic, oleic, linolenic, arachidonic, EPA, DHA, SFA, MUFA and PUFA. The ‘RP factor’ significantly affected the percentages of stearic, linoleic, linolenic, eicosatrienoic, arachidonic acids, SFA, and PUFA.

**Table 2.** Effects of glucomannan or glucomannan *plus* spirulina-RP on liver fatty acid profile (g/100 g of methyl ester) of cholesterol-fed fa/fa rats.

	Cholesterol addition	Control RP (C/HC)	Glucomannan-RP (G/HG)	Glucomannan *plus* Spirulin-RP (GS/HGS)	ANOVA		
					RP effect	Cholesterol effect	RP*Cholesterol interaction
Myristic (C14:0)	No	1.32 ± 0.53	1.51 ± 0.39	1.73 ± 0.67	0.41	0.28	0.71
	Yes	1.22 ± 0.08	1.44 ± 0.20	1.34 ± 0.30			
Palmitic (C16:0)	No	16.31 ± 1.31	19.05 ± 4.60	17.40 ± 3.22	0.059	0.010	0.75
	Yes	12.45 ± 2.38^a*^	16.91 ± 3.07^b^	13.17 ± 1.50^a*^			
Palmitoleic (C16:1)	No	20.40 ± 0.25	19.01 ± 3.27	20.78 ± 1.29	0.57	0.13	0.88
	Yes	22.67 ± 2.06	22.48 ± 2.35	23.30 ± 3.66			
Stearic (C18:0)	No	7.10 ± 1.29^a^	7.07 ± 1.19^a^	9.92 ± 1.68^b^	0.026	0.037	0.25
	Yes	6.32 ± 0.73	6.67 ± 0.85	7.27 ± 2.11**			
Oleic (C18:1n9)	No	31.96 ± 2.03^ab^	32.61 ± 1.64^a^	29.26 ± 2.40^b^	0.94	˂0.001	0.088
	Yes	38.50 ± 0.59**	38.62 ± 0.74***	41.12 ± 5.60**			
Linoleic (C18:2n6)	No	6.26 ± 1.31^a^	4.90 ± 0.35^b^	4.89 ± 0.44^b^	0.025	0.25	0.74
	Yes	6.29 ± 0.25	5.45 ± 0.84	5.51 ± 1.16			
γ-linolenic (C18:3n6)	No	0.21 ± 0.04	0.13 ± 0.04	0.22 ± 0.08	0.52	0.55	0.57
	Yes	0.17 ± 0.08	0.21 ± 0.07**	0.25 ± 0.22			
Eicosenoic (C20:1)	No	0.23 ± 0.10	0.19 ± 0.06	0.26 ± 0.22	0.43	0.50	0.86
	Yes	0.39 ± 0.10*	0.29 ± 0.15	0.44 ± 0.29			
Linolenic (C18:3n3)	No	0.27 ± 0.05	0.21 ± 0.03	0.26 ± 0.17	0.041	0.020	0.43
	Yes	0.41 ± 0.05^a**^	0.28 ± 0.05^b*^	0.46 ± 0.11^a^			
Eicosatrienoic (C20:3n6)	No	0.62 ± 0.12^a^	0.36 ± 0.14^b^	0.36 ± 0.14^b^	0.030	0.22	0.50
	Yes	0.64 ± 0.16^a^	0.52 ± 0.11^ab^	0.39 ± 0.11^b^			
Eicosatetraenoic (C20:4n6)	No	9.78 ± 0.76	9.56 ± 1.17	9.25 ± 0.76	0.001	˂0.001	0.010
	Yes	7.97 ± 0.84^a*^	5.16 ± 0.24^b**^	5.02 ± 0.94^b***^			
Eicosapentaenoic C20:5n3	No	2.11 ± 0.82	2.23 ± 0.49	2.75 ± 0.83	0.79	˂0.001	0.098
	Yes	1.06 ± 0.22^a*^	0.61 ± 0.37^ab**^	0.42 ± 0.29^b^			
Docosahexaenoic (C22:6n3)	No	3.45 ± 0.40	3.17 ± 0.84	2.92 ± 0.91	0.18	˂0.001	0.89
	Yes	1.91 ± 0.44**	1.35 ± 0.22**	1.32 ± 0.42*			
Saturated Fatty Acids	No	24.72 ± 1.53	27.63 ± 4.35	29.05 ± 3.67	0.031	˂0.001	0.29
	Yes	19.99 ± 2.18^a*^	25.02 ± 2.59^b^	21.77 ± 1.75^b*^			
Monosaturated Fatty Acids	No	52.58 ± 1.85	51.81 ± 2.66	50.30 ± 2.57	0.72	˂0.001	0.061
	Yes	61.56 ± 2.49^a***^	64.39 ± 2.23^b**^	64.86 ± 2.46^b***^			
Polyunsaturated Fatty Acids	No	22.70 ± 0.69	20.55 ± 2.35	20.65 ± 2.83	0.003	˂0.001	0.279
	Yes	18.45 ± 0.92***	13.59 ± 0.56***	13.37 ± 3.02**			

Values (mean ± SD, *n* = 6) in the same row bearing different letters were significantly different (*p *< 0.05; Bonferroni or T2-Tamhane tests). Values for the same diet but with different cholesterol content bearing asterisks were significantly different (*p < 0.05, **p < 0.01, ***p < 0.001). C/CH, control-RP with or without added cholesterol; G/HG, glucomannan-RP with or without added cholesterol; GS/HGS, glucomannan plus spirulina-RP with or without added cholesterol.

Palmitic acid (C16:0) percentage was significantly lower in HC *vs*. C and in HGS *vs*. GS rats but higher in HG *vs*. HC and HGS animals. Stearic acid (C18:0) percentage was lower in HGS *vs*. GS rats and in C and G *vs*. GS rats (*p *< 0.05). Oleic acid (C18:1n9) percentages were higher in HC, HG and HGS than in their C, G and GS counterparts. GS oleic percentage was lower in Gs vs. G rats. Linoleic acid (C18:2n6) percentage was lower in G and GS vs. C livers. Gamma-linolenic acid (C18:3n6) percentage was higher in HG *vs*. G. Eicosenoic acid (C20:1) was higher in HC *vs*. C rats. Linolenic acid (C18:3n3) percentages were lower in HG vs. HC and HGS groups; HC vs. C and HG vs. G livers presented higher linolenic acid percentages. Eicosatrienoic acid (C20:3n6) percentages were lower in G and GS *vs*. C livers and in HGS vs. HC liver. Arachidonic acid (C20:4n6) percentage was lower in HC, HG, HGS vs. C, G, GS livers and in HG and HGS vs. HC livers. Eicosapentaenoic acid (C20:5n3) percentage was significantly lower in HC and HG vs. C and in G livers, and in HGS vs. HC groups. Docosahexaenoic acid (C22:6n3) percentages were lower in HC, HG, HGS vs. C, G, GS livers.

SFA percentages were lower in HC and HGS *vs*. their C and GS counterparts’ livers and in HC *vs*. HG and HGS livers. MUFA percentages were higher in HC, HG, HGS *vs*. C, G, GS rats. The PUFA percentages were significantly lower in HC, HG and HGS *vs*. C, G, and GS.

Information regarding estimated desaturase and desaturase-elongase activity is shown in Table 3. The C20:4 n-6/C18:2 n-6 ratio and delta-5-desaturase activity were significantly affected by the type of RP* cholesterol interaction. None of the estimated activities were affected by 2RP effects; however, the cholesterol effect affected all activities except that of delta-6-desaturase. The C22:6n3/C18:3n3 ratio was lower in HC, HG and HGS *vs*. C, G, GS livers. The C20:4n6/C18:2n6 ratio was lower in HG and HGS *vs*. G and GS livers and in HG and HGS *vs*. HC livers. The C18:1n9/C18:0 ratio was higher in HG *vs*. G livers while SCD activity was higher in HC and HG *vs*. C and GS livers and in HC than in HG livers. Delta-5-desaturase activity was higher in G and GS livers *vs*. C livers.

Results in Table 4 show that liver redox indices (RI), GSH and GSSG were unaffected by the different diets.

**Table 3.** Effects of glucomannan or glucomannan *plus* spirulina-RP on liver fatty acid ratios (g/100 g of methyl ester) of cholesterol-fed fa/fa rats.

	Cholesterol addition	Control RP (C/HC)	Glucomannan-RP (G/HG)	Glucomannan *plus* Spirulin-RP (GS/HGS)	ANOVA		
					RP effect	Cholesterol effect	RP*Cholesterol interaction
C22:6n3/C18:3n3 ratio	No	12.99 ± 2.91	15.21 ± 2.27	12.47 ± 3.43	0.13	˂0.001	0.70
	Yes	4.73 ± 1.52^a**^	5.05 ± 1.89^a***^	2.85 ± 0.47^b***^			
C20:4n6/C18:2n6 ratio	No	1.62 ± 0.42	1.97 ± 0.32	1.90 ± 0.22	0.91	˂0.001	0.030
	Yes	1.27 ± 0.17^a^	0.96 ± 0.15^b***^	0.92 ± 0.09^b***^			
C16:1n7/C16:0 ratio	No	1.26 ± 0.10	1.08 ± 0.43	1.23 ± 0.24	0.15	0.002	0.63
	Yes	1.90 ± 0.38^a^*	1.38 ± 0.27^b^	1.77 ± 0.20^ab*^			
C18:1n9/C18:0 ratio	No	4.66 ± 1.11	4.73 ± 0.98	3.04 ± 0.73	0.21	˂0.001	0.22
	Yes	6.16 ± 0.73	5.86 ± 0.82*	5.99 ± 1.65			
Delta-5-desaturase	No	16.40 ± 3.30a	20.61 ± 5.79ab	28.22 ± 5.14a	0.10	˂0.001	0.046
	Yes	13.33 ± 5.08	10.30 ± 3.03**	13.63 ± 3.53**			
Delta-6-desaturase	No	0.034 ± 0.010	0.026 ± 0.010	0.044 ± 0.014	0.33	0.85	0.45
	Yes	0.027 ± 0.012	0.040 ± 0.016	0.041 ± 0.028			

Values (mean ± SD, *n* = 6) in the same row bearing different letters were significantly different (*p *< 0.05; Bonferroni or T2-Tamhane tests). Values for the same diet but with different cholesterol content bearing asterisks were significantly different (*p < 0.05, **p < 0.01, ***p < 0.001).

Stearoyl-CoA-desaturase, C16:1 n-7/C16:0; Delta-5-desaturase: C20:4 n-6/C20:3 n-6; Delta-6- desaturase: C18:3 n-6/C18:2 n-6. C/CH, control-RP with or without added cholesterol; G/HG, glucomannan-RP with or without added cholesterol; GS/HGS, glucomannan plus spirulina-RP with or without added cholesterol.

**Table 4.** Effects of glucomannan or glucomannan *plus* spirulina-RP on liver redox ratio, GSH and GSSG of cholesterol-fed fa/fa rats.

	Cholesterol addition	Control RP (C/HC)	Glucomannan-RP (G/HG)	Glucomannan *plus* Spirulina-RP (GS/HGS)	ANOVA		
					RP effect	Cholesterol effect	RP*Cholesterol interaction
GSH (µg/mg tissue)	No	0.60 ± 0.25	0.61 ± 0.22	0.52 ± 0.19	0.98	0.14	0.830
	Yes	0.50 ± 0.14	0.47 ± 0.12	0.52 ± 0.18			
GSSG (µg/mg tissue)	No	0.63 ± 0.22	0.55 ± 0.03	0.62 ± 0.18	0.70	0.27	0.875
	Yes	0.54 ± 0.06	0.51 ± 0.07	0.57 ± 0.15			
RI	No	0.48 ± 0.09	0.51 ± 0.10	0.45 ± 0.06	0.90	0.53	0.817
	Yes	0.47 ± 0.10	0.47 ± 0.09	0.48 ± 0.11			

Values (mean ± SD, n = 6) in the same row bearing different letters were significantly different (*p *< 0.05; Bonferroni or T2-Tamhane tests). Values for the same diet but with different cholesterol content bearing asterisks were significantly different (*p < 0.05, **p < 0.01, ***p < 0.001). GSH, reduced glutathione; GSSG, oxidized glutathione; RI, redox index; RI = [GSH/(GSH+GSSG)]. C/CH, control-RP with or without added cholesterol; G/HG, glucomannan-RP with or without added cholesterol; GS/HGS, glucomannan plus spirulina-RP with or without added cholesterol.

**Figure 2.**
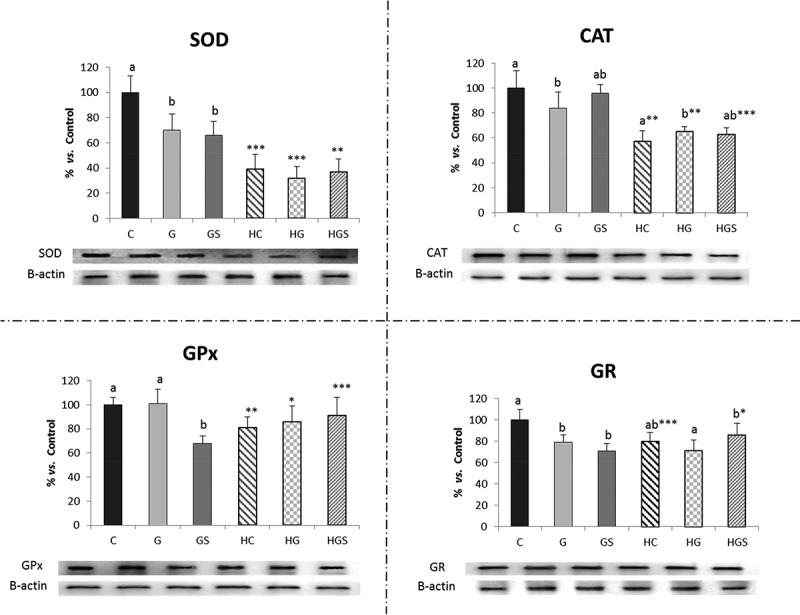
Protein levels of the main antioxidant enzymes measured by Western-blot in rats fed the glucomannan- and glucomannan *plus* spirulina-enriched pork experimental diets. Mean values and standard deviations (*n* = 6). Statistical comparison between HC *vs*. C, HG *vs*. G and HGS *vs*. GS groups, * indicate significant differences (*p < 0.05, **p < 0.01, ***p < 0.001). Bars with different letters indicate significant differences (at least *p *< 0.05) between C, G and GS or HC, HG and HGS groups. CAT, catalase; SOD, superoxide dismutase; GR, glutathione reductase; GPx, glutathione peroxidase. C/CH, control-RP with or without added cholesterol; G/HG, glucomannan-RP with or without added cholesterol; GS/HGS, glucomannan *plus* spirulina-RP with or without added cholesterol. Results were calculated as percentage relative to control.

Table 5 shows results of antioxidant enzyme activities and expressions. Enzyme activities were not significantly modified by the RP-type factor or the RP-type and cholesterol. GPx and CAT activity tended to low with cholesterol feeding. Western blot (WB) (Figure 2) shows that both factors and their interaction relevantly and significantly affect enzyme levels. The protein level (Western-blot) was significantly higher in all tested enzymes for HC *vs*. C rat livers. A significant decrease in CAT and SOD levels was observed in HG and HGS *vs*. G and GS rats. Spirulina addition significantly increase GPx and GR protein levels when compared HGS *vs*. GS.

**Table 5.** Effects of glucomannan or glucomannan *plus* spirulina-RP on liver antioxidant enzymes of cholesterol-fed fa/fa rats.

	Cholesterol addition	Control RP (C/HC)	Glucomannan-RP (G/HG)	Glucomannan *plus* Spirulina-RP (GS/HGS)	ANOVA		
					RP effect	Cholesterol effect	RP*Cholesterol interaction
Cu,Zn-SOD Expression (% vs. C)	No	100.0 ± 19.04	121.11 ± 30.47	95.32 ± 45.94	0.26	<0.001	0.41
	Yes	5.78 ± 3.27 ^a^***	5.14 ± 4.07 ^ab^**	0.35 ± 0.21 ^b^***			
Mn-SOD Expression (%vs.C)	No	100.0 ± 18.67 ^a^	44.30 ± 15.03 ^b^	52.91 ± 27.71 ^b^	<0.001	0.001	0.122
	Yes	183.2 ± 46.63 ^a^**	92.78 ± 61.20 ^b^	67.11 ± 10.42 ^b^			
SOD Activity (U/mg de protein)	No	0.14 ± 0.08	0.18 ± 0.08	0.16 ± 0.11	0.45	0.60	0.92
	Yes	0.16 ± 0.05	0.20 ± 0.06	0.16 ± 0.06			
CAT Expression (%vs. C)	No	100.0 ± 31.63 ^a^	60.28 ± 16.95 ^ab^	48.78 ± 18.02 ^b^	0.009	<0.001	0.010
	Yes	10.13 ± 6.22 ^ab^***	20.52 ± 9.02 ^a^***	3.99 ± 1.43 ^b^***			
CAT Activity (U/mg protein)	No	37.54 ± 7.94	33.91 ± 3.74	38.61 ± 11.82	0.87	0.061	0.79
	Yes	30.33 ± 7.53	30.41 ± 8.41	29.49 ± 13.51			
GPx Expression (% vs. C)	No	100.0 ± 27.53	96.45 ± 38.28	75.87 ± 25.48	0.085	<0.001	0.92
	Yes	23.67 ± 8.03^a^***	22.69 ± 10.46^a^***	7.39 ± 3.26^b^***			
GPx Activity (U/mg protein)	No	11952 ± 5986	9103 ± 2678	10952 ± 3011	0.47	0.006	0.72
	Yes	7364 ± 3072	6791 ± 1545	7307 ± 945			
GR Expression (% vs. C)	No	100.0 ± 34.42	100.5 ± 27.70	86.94 ± 23.05	0.14	<0.001	0.92
	Yes	31.60 ± 9.08^a^***	31.14 ± 15.90^a^***	11.56 ± 1.37^b^***			

Values (mean ± SD, *n* = 6) in the same row bearing different letters were significantly different (*p *< 0.05; Bonferroni or T2-Tamhane tests). Values for the same diet but with different cholesterol content bearing asterisks were significantly different (*p < 0.05, **p < 0.01, ***p < 0.001). C/CH, control-RP with or without added cholesterol; G/HG, glucomannan-RP with or without added cholesterol; GS/HGS, glucomannan plus spirulina-RP with or without added cholesterol.

Only CAT expression was significantly affected by RP-type and cholesterol interaction. RP-type significantly affected Mn-SOD and CAT expressions. Cholesterol significantly affected all enzyme expression. Cu/Zn-SOD expression in HGS *vs*. HC rats; Mn-SOD expression in GS *vs*. C and in HGS *vs*. HC rats were significantly lower; CAT expression in GS *vs*. C animals; and the GR and GPx expressions in HGS *vs*. HC rats. Glucomannan *plus* spirulina *vs*. glucomannan affected the CAT and GPx expression in HGS *vs*. HG rats.

Cu/Zn-SOD, CAT, GR and GPx were significantly lower in HC, HG and HGS *vs*. C, G and GS rats. Mn-SOD expression was significantly higher in HC *vs*. C rats (*p *< 0.01).

Several significant correlations were found. Liver weight and the hepatosomatic index correlated negatively and significantly with *Ldlr* expression, Cu/Zn-SOD, CAT, GPx and GR expression and WB levels, delta-6-elongase-desaturase and delta-5-desaturase activities (all *p *< 0.001) and GPx activity (at least *p *< 0.01) while a positive correlation was found with SCD and oleoyl-CoA reductase activities and Mn-SOD (at least *p *< 0.01).

## Discussion

This study shows for the first time that RP and cholesterol modified liver fatty acid profile and antioxidant enzyme activities, levels and expressions and *Ldlr* expression in fa/fa rats. These results were interrelated and also highly dependent on body weight. Zucker fa/fa rats became severely obese, their daily food intake was very high and they exhibited hypercholesterolemia as described by other others, very high daily intake and hypercholesterolemia in concurrence with other authors [35]. Surprisingly, G and GS included in RP did not have a satiating effect as has been suggested by other authors in relation to *Konjac glucomannan* [18,36]. In fact, comparison of slopes shows that for each kJ consumed, G and GS animals gained 0.002–0.005 g less than C rats. The stomach is known to produce ghrelin and leptin, two important food intake controllers. However, as fa/fa rats are leptin-resistant [37], any expected mechanism linking leptin and satiation has to be studied carefully. In addition, fructose and saturated fatty acids, two major compounds of the assayed diets, modify the production response of both gastric hormones [38]. In agreement with Beynen et al. [39], lower feed intake and growth were found in cholesterol-fed rats. As a result of this reduction, lower values of plasma glucose were found in the HC group. The higher plasma glucose and TyG values clearly suggest that diabetes and insulin resistance was pervasively present in all rat groups [40]. The TyG index was not significantly modified in HC when compared to C because of the mathematical proportion of the logarithm. The triglycerides are higher in HC due to cholesterol addition to diet, while glucose is lower because the food intake reduction.

All of the non-cholesterol and cholesterol-fed fa/fa rats were diabetic as their plasma glucose surpassed the cut-off point of 7 mmol/L, diabetes cut-off point for humans [41]. However, the prevalence of hyperglycemia >11.1 mmol/L was lower in groups that consumed cholesterol-added diets (50% in HC; 33% in HG and 17% in HGS rats). The presence of glucomannan and spirulina in the cholesterol-fed animals also reduced the TyG index with respect to their counterparts suggesting improvement of insulin resistance as TyG values of 9.1 and 10.3 have been proposed as the median and 95th percentile in patients affected by metabolic syndrome [40]. Rats consuming cholesterol-enriched diets showed lower weight gain and growth rates than their non-cholesterol counterparts. In addition, those rats presented lower total adipose tissue mass (data not shown), interestingly a positive significant correlation was found between plasma glucose and growth rate (*r* = 0.387; *p *= 0.024) and with total adipose tissue mass (*r* = 0.397; *p *= 0.020); explaining why cholesterol-fed animals glucose level improvement.

Data from a previous paper showed that the prevalence of severe cholesterolemia in non-cholesterol fa/fa rats was reduced by G-RP and GS-RP intake. The benefits of consuming G-RP and GS-RP were also much more evident as these meat products strongly limited the hypercholesterolemic effects of dietary cholesterol [42]. However, HG and HGS diets unexpectedly blocked *Ldlr* expression, thus the hypocholesterolemic effect of glucomannan or glucomannan *plus* spirulina appeared clearly unrelated with modifications in the *Ldlr* expression. Therefore it must be accepted that through fat retention by adsorption and/or the seizing of bile salts, glucomannan contributes to reducing the effects of high fat diets [16]. According to our data, 3 g/kg of spirulina present in HGS diets improved the hypocholesterolemic response of glucomannan in cholesterol-fed animals [43], probably by seizing bile salts as has been suggested in the case of some polyphenols [44]. Although we are far from knowing the mechanism involved in this expression inhibition, it can be suggested that it prevents additional liver cholesterol uptake (via lipoproteins) and steatosis.

As a consequence of their inner metabolism [33] and diet composition, C rats presented hypercholesterolemia [45] and have an increased percentage of oleic and palmitoleic acids in the liver in comparison to those reported by Viejo et al. [29] in control Wistar rats fed semisynthetic diets containing casein and olive oil. In addition, cholesterolemia and oleic acid appeared increased in cholesterol fed animals (HC, HG and HGS rats in comparison to C, G and GS rats). According to the ANOVA test the consumption of the hypercholesterolemic agent induced significant decrease on palmitic and steric acids and an increase on palmitoleic and oleic acids, their metabolic products. With respect to the comparison HC *vs*. C, both palmitic and stearic acid levels tended to decrease while palmitoleic and oleic acids to increase; clearly suggesting that the SCD activity (palmitoleic acid/palmitic acid and oleic acid/stearic acid ratios), a surrogate index of delta-9-desaturase activity, increased in HC *vs*. C animals. Viejo et al. [29] reported that oleic acid and the oleic/stearic acid ratio increased in the liver of rats fed cholesterol-enriched diets and suggested that this increase occurred in the form of cholesteryl oleate as a mechanism to reduce the free cholesterol pool in the liver, which in turn increases the activity of VLDL and LDL receptors in an attempt to decrease the plasma levels of the atherogenic VLDL and LDL particles.

HC, HG and HGS rats in comparison to C, G and GS rats have lower polyunsaturated fatty acids (total, arachidonic acid (AA), eicosapentaenoic acid (EPA) and docosahexaenoic acid (DHA)) while HC and HGS *vs*. C and GS also presented lower amounts of saturated fatty acid in the liver. In addition, arachidonic/linoleic acid (AA/AL) and docosahexaenoic/linolenic acid (DHA/LL) ratios were lower, suggesting that feeding fa/fa rats with cholesterol enriched experimental diets partially inhibited the delta-6-desaturase-elongase system, known to be responsible for transforming linoleic and linolenic acids into longer and more unsaturated fatty acids of the omega-6 and omega-3 families, respectively [20]. Bochenek and Rodgers [46] and Huang et al. [47] have described that delta-6-desaturase-elongase system activity diminishes in the presence of a cholesterol-enriched diet. Very recently, Jacobs et al. [24] suggested that delta-5-desaturase (D5D), delta-6-desaturase (D6D) and stearoyl-CoA desaturase (SCD) activities are related with the incidence of T2DM.

Our results suggest that SCD as liver fat increased due to cholesterol feeding. Elevated SCD activity in liver, estimated from total fatty acids, was positively related to liver fat histologically determined [48]. However, HG diet decreased the SCD activity observed in HC animals. According to [28], the SCD index should be preferably based on defined lipid fraction whereas SCD derived from total hepatic fatty acids should be cautiously interpreted. D5D activity was higher in GS *vs*. C rats suggesting that GS diet promoted anti-inflammatory properties [49]. In cholesterol fed rats, principally in diet HG and HGS *vs*. G and HGS, D5D activity decreased suggesting that, although glucomannan has a potent hypocholesterolemic effect, a side inflammatory effect should not be discarded. However, even though D6D was not significantly affected, a tendency to increase was also observed in those animals. The rise in D6D has been related to an increase in arachidonic acid which is a precursor from eicosanoids that can act as inflammatory mediators [6]. Unpublished histological data clearly suggest that inflammatory focal points were observed in the cholesterol-fa/fa rats.

The decrease in delta-6-desaturase-elongase observed in cholesterol-fed fa/fa rats was greater in HG and HGS animals vs. their G and GS counterparts. It is well known that linoleic and linolenic acids are less oxidizable than arachidonic acid, EPA and DHA. It can be speculated that partial inhibition of the transformation of linolenic acid in EPA and DHA and that of linoleic acid in arachidonic acid, could occur as a protective mechanism to avoid excess of highly peroxidable fatty acids and oxysterol in the liver.

Alterations in the regulation of the redox balance and an increase of reactive oxygen species (ROS) has been reported in animal models of diabetes [50]. The results in Table 4, expressed in µmol/g, showed GSH levels three times lower and GSSG levels three times higher than those reported for Wistar control rats by Bocanegra et al. [51]. The redox index of 0.60 found in the C group was also much lower than the one reported by Schultz et al. [52] for Wistar rats fed RP. This suggests that Zucker fa/fa rats have a genetic predisposition to develop diabetes, dyslipidemia and low redox index. The pre-existing antioxidant defense deficiency tended to be impaired after cholesterol feeding as reported by others in Wistar rats [51]. In diabetes the enzyme glucose-6-phosphate dehydrogenase (G6PD) is inhibited; therefore, the entire defense mechanism becomes compromised and lower amounts of enzymatic-cofactor NADPH are formed and available [50,53]. This cofactor is essential for GSH regeneration. The consumption of GS and G diets did not provide any change in the liver levels of GSH, GSSG and RI. Also, other studies have proved that dietary components, like fat or cholesterol affect the antioxidant defense in cells [54], leading to unexpected levels of non-enzymatic antioxidants (e.g. glutathione levels) and modifications of the enzymatic pathway. Our group have recently proved that elevated levels of dietary fat lead to high lipid peroxidation indirectly modifying GSH levels and glutathione enzyme activity [55].

In addition, in the groups that consumed cholesterol the RI, GSH and GSSG remained unchanged in relation to their non-cholesterol counterparts. With the exception of SOD, all other enzyme activities studied were negatively affected by cholesterol feeding. However, basal values of CAT activities were much lower while those of GPx were higher in this experiment than in a previous ones [52,56] suggesting that fa/fa rats address these enzyme activities to reduce the hydroperoxydes coming from PUFA. Once again, the relationship between delta-6-desaturase-enlogase and liver oxidation can be suggested. Our group has found that cholesterol-feeding greatly increased the absorption and bioavailability of iron (unpublished data) which is known to have pro-oxidant effects. Cordero Herrera et al. [57] reported that obese Zucker rats showed increased ROS production but no modification of GR or GPx enzymes with respect to lean Zucker rats.

Interestingly, in cholesterol-fed rats *vs*. non-cholesterol-fed ones, WB and PCR exhibited similar tendencies. The decrease in enzyme WB levels for GR was completely arrested in HG and HGS *vs*. G and GS rats; however, for most enzymes HG and overall HGS diets significantly blocked antioxidant enzyme expressions. Vázquez-Velasco et al. [17] also showed the negative effects of glucomannan or glucomannan *plus* spirulina-enriched surimies in fa/fa rats. Again, the negative effects of consuming glucomannan *plus* spirulina together with dietary cholesterol can be suggested.

However, our results clearly differ from those of Vázquez-Velasco et al. [17,18] which found much lower final body weight and cholesterolemia than in this study, both in non-cholesterol and cholesterol fed animals. In addition, liver size was not arrested by GHG and HGS diets as it was in the Vázquez-Velasco et al. studies [17,18] in both cholesterol or non-cholesterol groups. This suggests the importance of the matrix used for incorporating those ingredients.

Some important limitations of this paper are that only male growth fa/fa rats were used. Also, the study was performed with only one concentration of glucomannan or glucomannan *plus* spirulina added to RP. Future studies should assess potential benefits of different concentrations of glucomannan and glucomannan *plus* spirulina-RP and in a wider age range of fa/fa rats and their possible extrapolation to obese and dyslipidemic humans.

## Conclusion

RP with glucomannan or glucomannan/spirulina affect liver fatty acid profile and antioxidant status. Spirulina added to glucomannan tended to increase diet acceptability and D5D activity. Cholesterol feeding reduced delta-6-desaturase-elongase and D5D activities and increased SCD activity while markedly decreasing most antioxidant enzyme expression and levels and expression of *Ldlr* suggesting that the two mechanisms are linked. Although GHS arrested the hypercholesterolemic effect of dietary cholesterol observed and improved insulin resistance, given by the TyG index in HC animals, this diet negatively affected the delta-6-desaturase-elongase activity and markedly depressed the gene expression of antioxidant enzymes and *Ldlr* suggesting that caution needs to be taken when incorporating large amounts of glucomannan or glucomannan *plus* spirulina to cholesterol enriched diets.

## Supplementary Material

Supplementary Table

## Supplemental material

The supplemental data for this article can be accessed here.
