# Supplementary material for: Glucomannan- and glucomannan plus spirulina-enriched pork affect liver fatty acid profile, LDL receptor expression and antioxidant status in Zucker fa/fa rats fed atherogenic diets
Source: Food Nutr Res. 2016 Dec 20;61(1):1264710. doi: 10.1080/16546628.2017.1264710 (PMC5328336; doi:10.1080/16546628.2017.1264710)
Supplement: Supplementary Table [file zfnr_a_1264710_sm6874.docx]

**Glucomannan- and glucomannan plus spirulina-enriched pork affect liver fatty acid profile, LDL receptor expression and antioxidant status in Zucker fa/fa rats fed atherogenic diets**

**Supplementary Table**

Composition (g/kg) of the experimental diets^1^

|  | C diet | G diet | GS diet | HC diet | HG diet | HGS diet |
| --- | --- | --- | --- | --- | --- | --- |
| Cornstarch | 279.2 | 309.2 | 306.2 | 250 | 280 | 277.1 |
| Microcrystalline cellulose | 40.0 | 10.0 | 10.0 | 40.0 | 10.0 | 10.0 |
| Cholesterol | 0 | 0 | 0 | 24.3 | 24.3 | 24.2 |
| Cholic acid | 0 | 0 | 0 | 4.9 | 4.9 | 4.9 |
| Pork | 150.0 | 127.5 | 127.5 | 150 | 127.5 | 127.5 |
| Glucomannan | 0 | 22.5 | 22.5 | 0 | 22.5 | 22.5 |
| Spirulina | 0 | 0 | 3 | 0 | 0 | 3 |
| Energy content (MJ/kg diet) | 279.2 | 309.2 | 306.2 | 250.0 | 280.0 | 277.1 |

C: AIN-93M (85%) + Control pork meat (15%); G: AIN-93M (85%) + Glucomannan enriched pork meat (15%); GS: AIN-93M (85%) + Glucomannan *plus* spirulina enriched pork meat (15%). HC: AIN-93M (85%) + Control pork meat (15%) + hypercholesterolaemic agent (cholesterol and cholic acid); G: AIN-93M (85%) + Glucomannan enriched pork meat (15%) + hypercholesterolaemic agent (cholesterol and cholic acid); GS: AIN-93M (85%) + Glucomannan plus spirulina enriched pork meat (15%) + hypercholesterolaemic agent (cholesterol and cholic acid). ^1^Other ingredients (g/kg diet): casein. 127.5; palm olein. 140.0; dyetrose (carbohydrate composition: monosaccharides. 10; disaccharides. 40; trisaccharides. 50; tetrasaccharides and higher. 900). 131.75; sucrose. 85.0; choline bitartrate. 3.06; L-cystine. 1.53; t-butylhydroquinone. 0.03. Ain-93 M mineral mix #210050. 29.75 and Vitamin mix #310025. 12.16 (according to Reeves et al.. [23]).
